# Aortic Valve Replacement and Penicillin Desensitization in a Patient with *Abiotrophia defectiva* Aortic Valve Endocarditis

**DOI:** 10.1155/2021/1072049

**Published:** 2021-08-24

**Authors:** S. Kumar, S. Mahmood, A. Madras, A. Iyer

**Affiliations:** ^1^St. George Hospital, Sydney, Australia; ^2^Hornsby Hospital, Sydney, Australia; ^3^North Manchester General Hospital, Manchester, UK; ^4^Northern Beaches Hospital, Sydney, Australia

## Abstract

*Abiotrophia defectiva* is an uncommon and insidious yet destructive cause of infective endocarditis preferentially treated with penicillin/gentamicin and often requiring surgical treatment. A 60-year-old man with penicillin anaphylaxis history presented with fevers and a nonspecific constellation of symptoms. He was ultimately diagnosed with bicuspid aortic valve infective endocarditis based on blood cultures growing *A.defectiva* and echocardiographic evidence of bicuspid aortic valve, severe valvular regurgitation, and 5 × 7 mm vegetation. Aortic valve replacement and culture yielded penicillin-sensitive *A.defectiva*. After successful penicillin desensitization, antibiotic therapy was switched from vancomycin/gentamicin to benzylpenicillin. This is the first published case of penicillin desensitization in a patient with *A.defectiva*-associated infection. Penicillin desensitization, optimal antibiotic therapy, prompt aortic valve replacement, and close collaboration between cardiology and various other specialties were essential in achieving a positive outcome.

## 1. Introduction

*Abiotrophia defectiva* is a coccobacillus bacterium which is part of the normal flora of the intestinal tract, oral cavity, and urogenital tract [1]. *A.defectiva* has been reported to cause various deep-seated infections such as bacteremia, endocarditis, osteomyelitis, and endophthalmitis [2–6]. *A.defectiva* is an uncommon (<1%) but aggressive and potentially devastating cause of infective endocarditis with high rates of complications, surgery, treatment failure, and mortality [2, 7]. Penicillin is the preferred antibiotic therapy and patients often require surgical valvular replacement. We present the case of a 60-year-old man with fevers and nonspecific symptoms diagnosed with bicuspid aortic valve, *A.defectiva* bacteraemia, and aortic valve infective endocarditis with severe aortic regurgitation at presentation necessitating surgical aortic valve replacement. This is the first reported case where penicillin desensitization was utilized in a patient with *A.defectiva*-associated infection to facilitate optimal antimicrobial therapy. Written informed consent was obtained from the patient for publication.

## 2. Case Presentation

A 60-year-old man was referred to hospital by his general practitioner with fevers. He had 3-month history of lower back pain, night sweats, fatigue, and 13-kilogram weight loss. He had no significant past medical history or recent dental procedures, denied intravenous drug use, and was not taking any regular medications (including immunosuppressants). He reported a history of anaphylaxis to penicillin at 6 months of age.

On examination, he was tachycardic (102 bpm), normotensive (153/64 mmHg), and afebrile. He exhibited a grade 3/6 diastolic murmur in the aortic region and midlumbar spinal tenderness. He lacked signs of cardiac failure, neurological deficits, or peripheral stigmata of infective endocarditis. Systems review did not reveal any other infective source for his fevers. There was no clinical lymphadenopathy, abdominal masses, or organomegaly or palpable purpura. Twelve lead electrocardiogram (ECG) demonstrated sinus tachycardia.

Initial biochemistry (Table 1) revealed elevated C-reactive protein (CRP) and erythrocyte sedimentation rate (ESR), mild renal impairment, hypoalbuminemia, and normocytic anemia. Inpatient blood culture yielded gram-positive coccobacilli in aerobic and anaerobic bottles after 18 hours of incubation deemed to be *A.defectiva*. Transthoracic echocardiogram (TTE) showed bicuspid aortic valve with moderate regurgitation and small vegetation, normal biventricular size and function, and mildly dilated ascending aorta (3.9 cm). *A. defectiva* was sensitive to vancomycin but not to penicillin (mean inhibitory concentration (MIC) 0.25-2.0 *μ*g/mL). Three subsequent blood cultures were negative. Viral hepatitis, tuberculosis, human immunodeficiency virus (HIV), vasculitis, and malignancy screens were negative.

**Figure 1:**
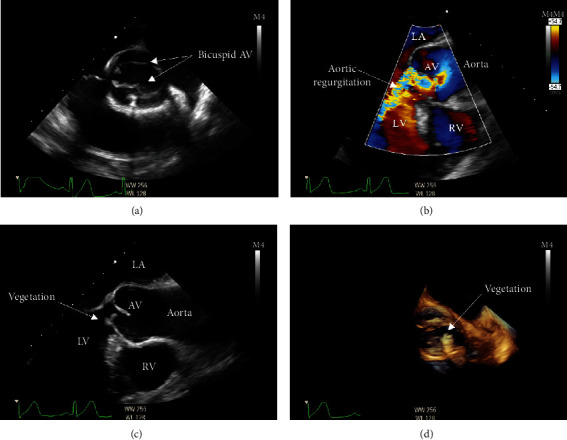
Transoesophageal echocardiogram images suggestive of bicuspid native aortic valve infective endocarditis. AV: aortic valve; LV: left ventricle; RV: right ventricle; LA: left atrium. Transoesophageal echocardiogram demonstrates the classical “fish mouth” appearance of aortic bicuspid valve during systole (a), severe aortic regurgitation (b), 5 × 7 mm vegetation adherent to the ventricular aspect of the aortic valve seen in two-dimensional echocardiogram (c), and three-dimensional echocardiogram (d).

**Figure 2:**
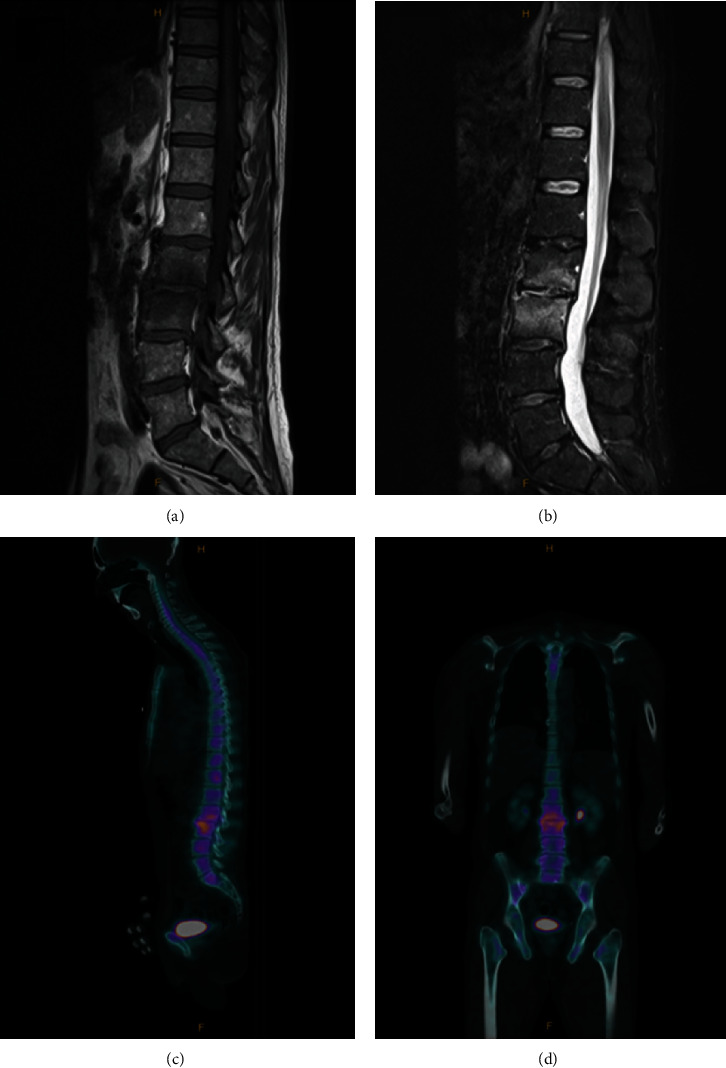
Magnetic resonance imaging (MRI) and single-photon emission computed tomography-computed tomography (SPECT-CT) images of the spine suggestive of L3/L4 discitis. Sagittal-view MRI whole spine demonstrates hypointense T1-weighted signal (a) and hyperintense short tau inversion recovery- (STIR-) weighted signal (b) surrounding the L3/L4 vertebral disc consistent with edema, with no evidence of collection. SPECT-CT whole spine shows increased uptake surrounding the L3/L4 vertebral disc in both sagittal view and coronal view consistent with L3/L4 discitis.

Transoesophageal echocardiogram (TOE) revealed severe aortic regurgitation with significant destruction and a 5 × 7 mm vegetation (Figure 1). Magnetic resonance imaging (MRI) scan of the whole spine and bone scan localized with single photon emission computed tomography (SPECT)/CT both suggested L3/L4 discitis (Figure 2). Orthopantomogram did not yield an abscess or dental caries. Cardiothoracic surgical referral prompted CT angiograms of coronary arteries (calcium score 0, no coronary artery disease) and thoracic aorta (4.2 cm ascending aorta aneurysm).

Empiric treatment of intravenous cephazolin (2 g three times daily), vancomycin 1.5 g twice daily, and gentamicin 80 mg twice daily was recommended by infectious diseases. On day 16 of admission, he underwent bioprosthetic aortic valve replacement followed by routine 48-hour intensive care admission. At 1 week postoperatively, TTE showed a well-seated and functioning bioprosthetic aortic valve with no vegetations. Aortic valve culture grew *A.defectiva* sensitive to penicillin (MIC 0.016 *μ*g/mL). As per infectious and allergy specialist recommendations, he underwent oral desensitization to penicillin on postoperative day 7 in intensive care. He then switched to intravenous benzylpenicillin 1.8 g four hourly for 6 weeks from operation date, completed by 3 months of oral phenoxymethylpenicillin 750 mg six hourly.

At treatment completion, he regained 9 kilograms of weight and CRP normalized. He will follow up with an allergy specialist for penicillin skin testing to guide future endocarditis prophylaxis.

## 3. Discussion

*Abiotrophia defectiva* is an uncommon (<1%) yet aggressive cause of infective endocarditis with high rates of complications, surgery, and mortality [2, 7]. Patients experience fever, fatigue, and sometimes significant weight loss for a median 3 months prior to diagnosis [2]. Complications are frequent at presentation, such as valvular regurgitation, heart failure, and septic embolization, with surgery indicated in >50% of cases [2]. Delays in diagnosis occur due to the insidious and nonspecific nature of symptoms and difficulties in culturing the bacteria with fastidious growth requirements and gram-stain pleomorphism [2, 7]. Similarly, our patient had undiagnosed subacute infection for 3 months during which his aortic valve was destroyed to the point of severe regurgitation. *A.defectiva* has a particular predilection for abnormal aortic and mitral valves [2, 7] due to its moderate ability to bind fibronectin, an extracellular matrix protein that acts as a receptor on valvular endothelium [7]. Once diagnosed, treatment failure is common due to emerging penicillin resistance [8] and inadequate response to antibiotics that demonstrate in-vitro sensitivity [7]. In our case, despite 2 weeks of culture- and sensitivity-guided vancomycin therapy, his aortic valve culture still grew *A.defectiva*. Delayed diagnosis and treatment failure often necessitate urgent surgical valvular replacement due to valvular regurgitation causing heart failure [2, 7]. On presentation, despite our patient having severe aortic regurgitation, the vegetation was not large, and he lacked evidence of heart failure and so did not meet criteria for emergency surgery. However, given the destructive nature of *A.defectiva*, we proceeded with aortic valve replacement after a period of stabilization with intravenous antibiotics.

Penicillin is the most common cause of drug-induced anaphylaxis [9]. Penicillin desensitization is indicated in patients with penicillin anaphylaxis history when penicillin is the preferred antibiotic [9, 10]. Penicillin is first-line therapy for *A.defectiva* endocarditis [10], although due to intermediate penicillin-sensitivity, desensitization was not pursued initially. However, given aortic valve culture that showed penicillin-sensitive *A.defectiva* and was still positive despite 2 weeks of vancomycin, we performed oral desensitization in consultation with infectious diseases and local protocols (Table 2) [10]. Due to his new bioprosthetic valve and history of endocarditis, he is at high risk of future endocarditis and will require antibiotic prophylaxis prior to high-risk dental procedures [10]. Desensitization develops short-term tolerance however does not determine the presence of true allergy [9] and therefore, he will follow up with an allergy specialist for penicillin skin testing to determine if ampicillin can be used for future endocarditis prophylaxis.

## 4. Conclusion

*Abiotrophia defectiva* is an uncommon yet important and potentially destructive cause of subacute infective endocarditis and presents insidiously and nonspecifically, which can lead to delayed diagnosis and significant complications at presentation. Preferred antibiotic treatment is with penicillin/gentamicin, and surgical treatment is often required. We have described the first reported case of penicillin desensitization in *A.defectiva* endocarditis management. Valve culture proved a useful adjunct to blood culture testing in this patient and led to a meaningful change in management. Penicillin desensitization (which facilitated appropriate antibiotic therapy), prompt aortic valve replacement, and close collaboration between multiple specialties including cardiologists, cardiothoracic surgeons, intensivists, infectious, allergy, and microbiology specialists were essential in achieving a positive outcome. Cardiologists and other physicians should be made more aware of *A.defectiva* as a cause for subacute endocarditis and the need for earlier diagnosis and treatment to reduce the high rates of complications, surgery, and mortality.

## Figures and Tables

**Table 1:** Initial results of serum laboratory investigations.

Study	Result	Normal range (units)
C-reactive protein	88.1^H^	<3 (mg/L)
Erythrocyte sedimentation rate	95^H^	<21 (mm/h)
White cell count	7.7	4-11 (×10^9^/L)
Neutrophils	6.2	2-8 (×10^9^/L)
Platelets	302	150-450 (×10^9^/L)
Hemoglobin	105^L^	130-180 (g/L)
Mean corpuscular volume	91	80-99 (fL)
Creatinine	111^H^	60-110 (*μ*mol/L)
Urea	7.5	3-9 (mmol/L)
Estimated glomerular filtration rate	62	>59 (mL/min/1.73m^2^)
Bicarbonate	28	22-32 (mmol/L)
Albumin	28^L^	35-50 (g/L)
Corrected calcium	2.47	2.15-2.55 (mmol/L)
Phosphate	1.05	0.75-1.50 (mmol/L)
Thyroid-stimulating hormone	0.79	0.50-5.00 (mIU/L)
Free thyroxine	22.4	9-25 (pmol/L)

H: high; L: low. Initial full blood count and biochemistry revealed elevated inflammatory markers such as C-reactive protein and erythrocyte sedimentation rate. Other abnormalities included mild renal impairment, hypoalbuminemia, and normocytic anemia, all likely secondary to subacute infection. Thyroid function was normal.

**Table 2:** Rapid oral phenoxymethylpenicillin desensitization protocol for adults.

Step (at 15-minute intervals)	Phenoxymethylpenicillin suspension (mg/mL)	Volume (mL)	Dose (mg)	Cumulative dose (mg)
1	0.5	0.1	0.05	0.05
2	0.5	0.2	0.1	0.15
3	0.5	0.4	0.2	0.35
4	0.5	0.8	0.4	0.75
5	0.5	1.6	0.8	1.55
6	0.5	3.2	1.6	3.15
7	0.5	6.4	3.2	6.35
8	5.0	1.2	6	12.35
9	5.0	2.4	12	24.35
10	5.0	4.8	24	48.35
11	50	1	50	98.35
12	50	2	100	198.35
13	50	4	200	398.35
14	50	8	400	798.35
15	Observe for 30 minutes; if no reaction occurs, administer phenoxymethylpenicillin 500 mg orally or benzylpenicillin 1.2 g intravenously. Start therapy with the required drug at the standard dosage for the indication.			

Source: eTG complete by Therapeutic Guidelines [Internet]. Melbourne (Vic):Therapeutic Guidelines Ltd.; 2020, available at https://tgldcdp.tg.org.au/etgcomplete accessed April 18, 2020.

## Data Availability

Deidentified data supporting the conclusions of this case report can be requested by contacting the corresponding author, Dr. Shejil Kumar.

## References

[1] Christensen J. J., Facklam R. R. (2001). Granulicatella and Abiotrophia species from human clinical specimens. *Journal of Clinical Microbiology*.

[2] Téllez A., Ambrosioni J., Llopis J. (2018). Epidemiology, clinical features, and outcome of infective endocarditis due to Abiotrophia species and Granulicatella species: report of 76 cases, 2000-2015. *Clinical Infectious Diseases*.

[3] Woo P. C., Fung A. M., Lau S. K. (2003). Granulicatella adiacens and Abiotrophia defectiva bacteraemia characterized by 16S rRNA gene sequencing. *Journal of Medical Microbiology*.

[4] Alberti M. O., Hindler J. A., Humphries R. M. (2015). Antimicrobial susceptibilities of Abiotrophia defectiva, Granulicatella adiacens, and Granulicatella elegans. *Antimicrobial Agents and Chemotherapy*.

[5] Puzzolante C., Cuomo G., Meschiari M. (2019). Granulicatella adiacens and Abiotrophia defectiva Native Vertebral Osteomyelitis: Three Cases and Literature Review of Clinical Characteristics and Treatment Approach. *Case Reports in Infectious Diseases*.

[6] Horstkotte M. A., Dobinsky S., Rohde H. (2010). Abiotrophia defectiva endophthalmitis with retinal involvement and infiltrative keratitis: case report and review of the literature. *European Journal of Clinical Microbiology & Infectious Diseases*.

[7] Carleo M. A., del Giudice A., Viglietti R., Rosario P., Esposito V. (2015). Aortic valve endocarditis caused by *Abiotrophia defectiva*: case report and literature overview. *In Vivo*.

[8] Prasidthrathsint K., Fisher M. A. (2017). Antimicrobial susceptibility patterns among a large, Nationwide cohort of Abiotrophia and Granulicatella clinical isolates. *Journal of Clinical Microbiology*.

[9] Reynoso D., White A. C. (2020). Penicillin allergy. *The New England Journal of Medicine*.

[10] eTG complete by Therapeutic Guidelines [Internet] Melbourne (Vic):Therapeutic Guidelines Ltd. https://tgldcdp.tg.org.au/etgcomplete.

